# Advanced diagnostic methods for nontuberculous mycobacterial infections

**DOI:** 10.3389/ftubr.2026.1760581

**Published:** 2026-03-25

**Authors:** Sneha Singh, Amresh Kumar Singh, Sushil Kumar, Nandini Singh, Ashwini Kumar Mishra, Aroop Mohanty

**Affiliations:** 1 Departments of Zoology and Environmental Sciences, Deen Dayal Upadhyaya Gorakhpur University, Gorakhpur, Uttar Pradesh, India; 2 Department of Microbiology, Baba Raghav Das Medical College, Gorakhpur, Uttar Pradesh, India; 3 Department of Zoology, Deen Dayal Upadhyaya Gorakhpur University, Gorakhpur, Uttar Pradesh, India; 4 Department of Tuberculosis & Chest, Baba Raghav Das Medical College, Gorakhpur, Uttar Pradesh, India; 5 Department of Microbiology, All India Institute of Medical Sciences, Gorakhpur, Uttar Pradesh, India

**Keywords:** diagnosis of NTM, *M. avium* complex, non-tuberculous mycobacteria, sequencing, tuberculosis

## Abstract

Nontuberculous mycobacteria (NTM) represent an increasingly significant cause of pulmonary and extrapulmonary infections, but are sometimes misinterpreted as tuberculosis (TB) owing to overlapping clinical and microbiological characteristics. Conventional diagnostic approaches, such as Ziehl-Neelsen staining and culture in a Mycobacterial Growth Indicator Tube (MGIT) system, are constrained by extended incubation times, are insufficient for accurate species differentiation, and are limited by prolonged incubation periods. Recent molecular and genomic advances have transformed NTM diagnostics by enabling rapid, specific, and high-resolution identification. Line probe assays (e.g., GenoType Mycobacterium CM/AS assay) and multiplex PCR have enhanced the ability to distinguish between NTM species such as *Mycobacterium absessus, M. fortuitum*, and *M. avium* complex and *M. tuberculosis* complex, which is essential for proper treatment and epidemiological mapping. Among newer proteomic platforms, matrix-assisted laser desorption/ionization time-of-flight (MALDI-TOF) mass spectrometry has emerged as a transformative, cost-effective technology capable of identifying *Mycobacterium* species directly from culture isolates through protein fingerprinting. It provides rapid, reproducible, and highly discriminatory identification between closely related species. Next-generation sequencing (NGS) and whole genome sequencing approaches now offer unprecedented insight into species identification, strain typing, and drug-resistance prediction, complementing traditional culture-based susceptibility testing. Newer techniques such as metagenomics NGS (mNGS), targeted NGS (tNGS) multilocus sequence typing, and mycobacterial interspersed repetitive unit-variable number tandem repeats **(**MIRU-VNTR) genotyping facilitate subspecies-level resolution and real-time outbreak surveillance. Moreover, molecular beacons, insertion sequence analysis, and repetitive sequence-based polymerase chain reaction (Rep-PCR) enhance detection sensitivity even in paucibacillary samples. The integration of genomic data with automated diagnostic system promises earlier intervention, accurate species delineation, and improved patient outcome.

## 1 Introduction

Two major mycobacterial infections are *Mycobacterium (M.) tuberculosis* and *M. leprae*, which cause tuberculosis and leprosy, respectively. In spite of these, nontuberculous mycobacteria (NTM) is a mixed group of many species, such as *M. avium* complex (MAC), *M. kansasii, M. abscessus, M. szulgai* and *M. ulcerans*, and is accountable for rising infection rates worldwide (1). NTM are ubiquitous mycobacteria found in the environment, particularly in contaminated water, soil, dust, biofilms, aerosols, and hospital-based equipment responsible for affecting patients with immunosuppression or structural lung disease, and can frequently impact skin, the lymph nodes, or the lungs (2).

NTM was once believed to be an ambient organism; however, eventually it was recognized as a possible opportunistic pathogen linked to extrapulmonary and pulmonary infection (3, 4). NTM prevalence, mostly with pulmonary infections, is rising globally. Some studies show an increase in NTM infection in the United States from 8.2 to 20 per 100,000 persons (1997–2020) and in Hawaii from 20 to 44 per 100,000 persons (5, 6). Globally, NTM isolation increases ~4% per year, with MAC being the most common species. Among cystic fibrosis patients, the prevalence of NTM infection has been reported to be approximately 7.9%. In contrast, studies from African regions report lower prevalence rates, although the exact values vary depending on the population studied and diagnostic methods used. The prevalence of NTM infections varies geographically, with higher rates reported in East Asian countries such as Japan and South Korea, where isolation rates range between 13% and 15% (6, 7). In Asia, the disease is often linked to that involving MAC and *M. abscessus*. NTM pulmonary disease (NTM-PD) prevalence is around 1.1% in India (8). However, NTM isolation in India is highly variable, and commonly mentioned ranges are approximately 0.7%−34% depending on the region and patient population (9).

NTM bacteria are found frequently in environments that makes it very hard to identify its infections. Regarding diagnosis, clinicians face major problems due to widespread presence of these bacteria in nature. The major problems when finding NTM infections are to distinguish between contamination and real infection, as there are symptoms that look like tuberculosis, and it can be difficult to find the exact species (10). There is not a standard approach to diagnose disease outside of the lungs; smear tests have limits, and biofilm growth makes detection less accurate. As per laboratory findings, detecting NTM in respiratory samples does not always mean it is causing lung disease because there is also the possibility of normal presence, temporary infection, or sample contamination. We also see that when NTM is found in clean human body parts, such as blood, tissues, brain fluid, or chest fluid, it means the infection is real and needs treatment (11). This suggests that conventional and molecular techniques approach are required for the identification of NTM species in just a few hours. NTM species display wide inter- and intra-species variability in drug susceptibility, making standardized diagnostic and treatment approaches challenging and highlighting the necessity for species-level drug sensitivity testing (DST). Natural resistance, inducible resistance, and mutational resistance are developed by inadequate drug exposure and selection of drugs in a very complicated way and is measured by DST. Different medications used to treat NTM disease have different roles for these three drug susceptibility determinants. The selection and optimization of medication treatment regimens depends on an understanding of their relative use (1).

NTM disease exerts a significant economic strain on individuals and healthcare systems due to extended diagnostic delays, protracted treatment periods, costly medication protocols, and recurrent hospitalization. Compared to tuberculosis therapy, the treatment of NTM pulmonary disease usually requires the use of several antibiotics for 12–18 months or longer, which substantially increases the expense. Based on these estimations, the annual healthcare costs for patients with NTM pulmonary disease in the United States are between US$14,000 and US$36,000 more than those of people without NTM infection (12). Based on a Korean study, each patient's yearly direct medical expenses range from US$3,500 to US$10,000, mostly as a result of long-term antibiotic treatment and follow-up (13). Furthermore, because of complicated treatment plans and higher hospitalization rates, macrolide-resistant NTM cases are linked to even higher expenditures. A considerable indirect social and economic effect results from the disease's effects on productivity, employment capability, and quality of life in addition to direct medical expenses.

Treatment of NTM diseases remains challenging due to the diverse antimicrobial susceptibility patterns and complex and species-specific treatment regimens, compounded by limited literature on advanced diagnostic methods. Our article systematically evaluates the current understanding of advanced diagnostic approaches and drug resistance in NTM. The objectives of this article are to highlight that multiple clinical, microbiological, and methodological factors collectively shape the diagnostic outcomes and to emphasize the need for individualized treatment strategies, standardized diagnostic approaches, and optimized antibiotic management to prevent emerging NTM infections.

## 2 Major pathogenic NTM

Major pathogenic NTM infections are characterized by their environmental resilience, biofilm-forming capacity, drug resistance, and association with chronic or disseminated disease, particularly in older adults, immunocompromised individuals, and those with lung abnormalities. NTM comprise a diverse group of environmental mycobacteria, but only a subset is strongly associated with human disease, and MAC remains the most prominent pathogenic cluster worldwide. MAC species, especially *M. avium, M. intracellulare*, and *M. chimera*, are widely documented as the predominant cause of NTM pulmonary disease, specifically in immunocompromised hosts (14). *M. chimera* has been implicated in invasive infections linked to contaminated heater–cooler units (15). The *M. abscessus* complex (MAB), including subspecies *abscessus, bolletii*, and *massiliense*, are increasingly significant pathogens due to its intrinsic and inducible macrolide resistance, leading to poor treatment outcome (16). MAB is particularly associated with chronic lung disease, postsurgical infections, and outbreaks linked to contaminated medical devices.

Other important slow-growing pathogens include *M. kansasii, M. xenopi, M. malmoense, M. simiae*, and *M. szulgai*, each linked with regional disease burden including in India (14). Recent genomic analyses have also highlighted *M. gordonae, M. paragordonae*, and *M. saskatchewanense* emerging as significant environmental isolates, especially in hospital waste water systems (17). Among rapid growers, *M. fortuitum, M. chelonae*, and *M. mucogenicum* commonly cause skin and soft-tissue infections, catheter-associated infections, and postsurgical complications, respectively (2). These species are also frequently implicated in healthcare-associated outbreaks due to their ability to survive disinfectants and form biofilms inside water systems (18). It has been estimated that, globally, *M. intracellularae, M. avium, M. abscessus*, and *M. fortuitum* are among the most prevalent NTM species in clinical isolates across Asia, Europe, and North America. A limited number of NTM species are responsible for many clinically significant and drug-resistant infections (19). Among these, *M. abscessus*, MAC, and *M. kansasii* are most frequently associated with treatment failure due to intrinsic or acquired antimicrobial resistance. *M. abscessus* is recognized as one of the most drug-resistant NTMs, posing substantial therapeutic and diagnostic challenges (1).

## 3 Clinical presentation of NTM

NTM causes a wide spectrum of clinical disease and is the most common presentation and is typically characterized by chronic cough, sputum production, dyspnea, fatigue, weight loss, and occasional hemoptysis (20). Radiologically, nodular/bronchiectatic disease with tree-in-bud opacities and bronchiectasis is characteristic of MAC infection (21). Patients with underlying lung disorders—bronchiectasis, COPD, cystic fibrosis, and prior TB—are highly predisposed to NTM-PD, and these conditions worsen the prognosis (22, 23). It has also been reported that cervical lymphadenitis, recurrent fever, and multi-organ involvement define disseminated NTM, and mortality is significantly higher among patients above 65 years of age (24, 25). A non-infectious immunological reaction can be caused by inhalation of aerosolized NTM from hot tubs, pools, or humidifiers and is often linked to MAC or other waterborne NTM (26).

### 3.1 Pulmonary NTM disease (NTM-PD)

This is the most common form of the disease, accounting for nearly 80%−90% of all NTM infections. It presents with chronic cough, sputum production, breathlessness, weight loss, fatigue, and radiological features such as nodular-bronchiectasis disease or cavitation. It is mainly caused by *Mycobacterium avium complex* (MAC), *M. abscessus*, and M. *kansasii* (20, 21).

### 3.2 Extrapulmonary NTM disease

**Figure 1:**
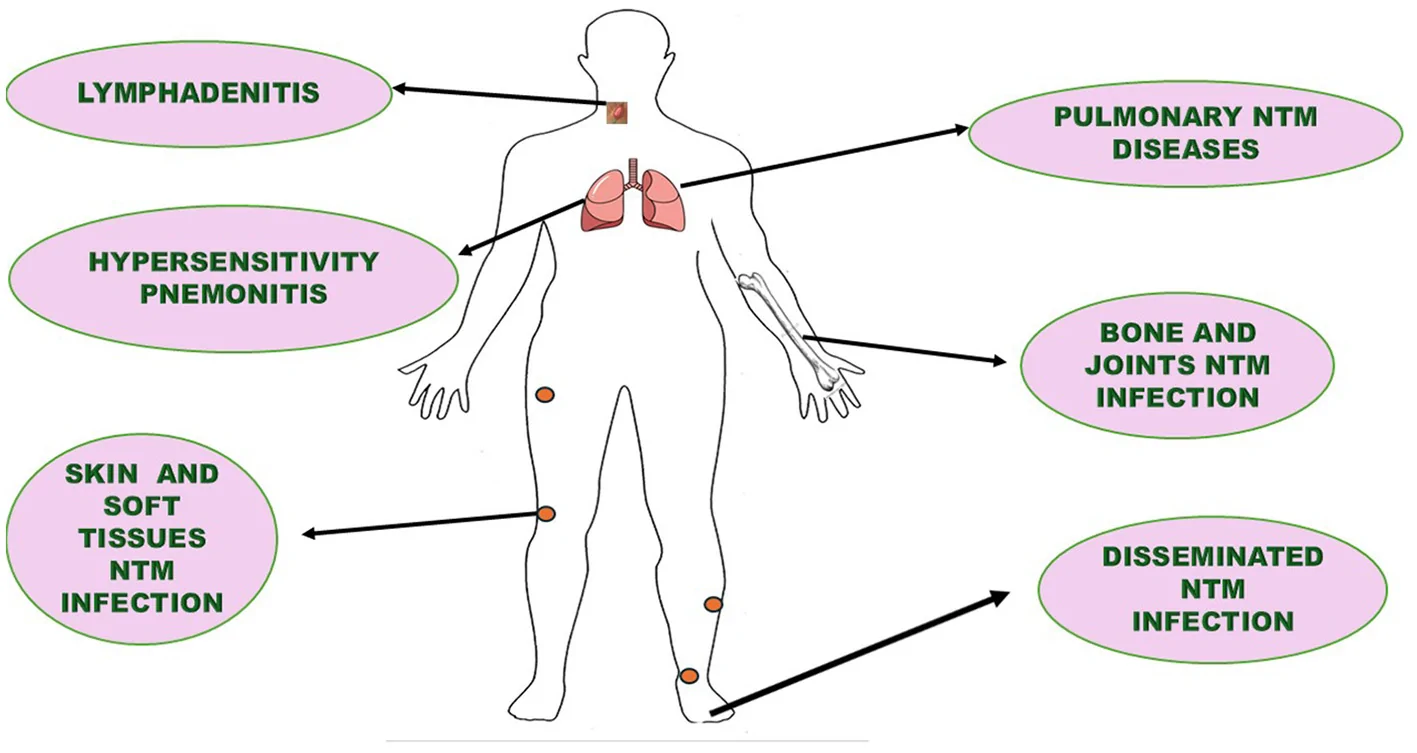
Clinical spectrum of nontuberculous mycobacterial (NTM) infections in humans. This figure illustrates the major anatomical sites and different disease manifestations associated with NTM. Pulmonary involvement is the most common presentation, while extrapulmonary disease includes lymphadenitis, skin and soft-tissue infections, and bone and joint infections. Severe manifestations such as disseminated NTM disease and hypersensitivity pneumonitis (“hot-tub lung”) are also depicted (2, 20, 21, 27).

Extrapulmonary NTM disease can involve multiple organs and tissues outside the lungs. Cervical lymphadenitis is a common manifestation, particularly affecting cervical lymph nodes in children (Figure 1). Skin and soft-tissue infections may occur following trauma, surgery, or injections and are most commonly caused by rapidly growing mycobacteria such as *M. abscessus, M. chelonae*, and *M. fortuitum* (27). NTM can also lead to bone and joint infections, including osteomyelitis and septic arthritis (28). Disseminated NTM disease is primarily observed in immunocompromised individuals, including patients with AIDS or those with anti-IFN-γ autoantibodies, and is characterized by fever, lymphadenitis, hepatosplenomegaly, bacteremia, and multi-organ involvement, with slow-growing NTM such as MAC being common causes (3). In addition, hypersensitivity pneumonitis (“hot-tub lung”) represents a non-infectious immunological reaction caused by inhalation of aerosolized NTM from hot tubs, pools, or humidifiers, and is often associated with MAC or other waterborne NTM (26).

## 4 Conventional diagnostic tools for NTM

**Figure 2:**
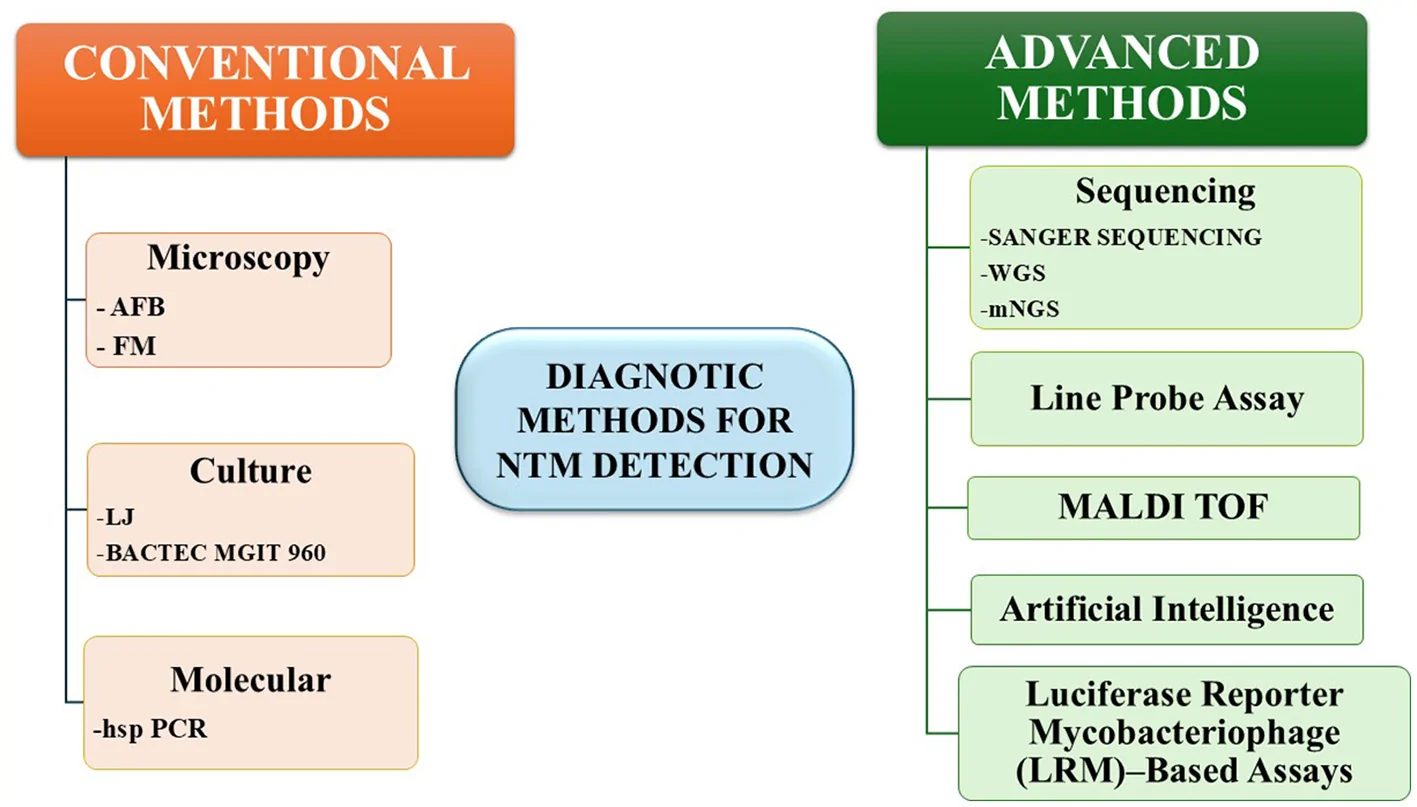
Diagnostic methods for the detection of nontuberculous mycobacteria (NTM). The figure summarizes the major conventional and advanced diagnostic approaches used for NTM detection. Conventional methods include microscopy (AFB, FM), culture (LJ, BACTEC MGIT 960), and basic molecular assays, whereas advanced approaches include sequencing technologies (Sanger sequencing, WGS, mNGS), line probe assays, MALDI-TOF mass spectrometry, artificial intelligence–based tools, and luciferase reporter mycobacteriophage–based assays. AFB, acid-fast bacilli; FM, fluorescence microscopy; LJ, Löwenstein–Jensen medium; MGIT 960, mycobacteria growth indicator tube 960 system; hsp PCR, heat-shock protein gene–based polymerase chain reaction; WGS, whole genome sequencing; mNGS, metagenomic next-generation sequencing; MALDI-TOF, matrix-assisted laser desorption/ionization–time-of-flight; AI, artificial intelligence; MIRU-VNTR, mycobacterial interspersed repetitive unit-variable number of tandem repeat.

Traditional (conventional) tests for finding NTM bacteria use basic lab methods that recognize acid-fast NTM and try to identify the different species. Clinicians prefer to use methods with Ziehl-Neelsen (ZN) or Kinyoun staining, followed by culture methods with solid media such as Löwenstein–Jensen (LJ) or in liquid systems such as MGIT (Figure 2). Other standard tools include Hsp65 gene-based identification. These standard methods are helpful, but they take too much time and are not very accurate for identifying the right species. However, the detailed methodology with their utilization is mentioned here.

### 4.1 Microscopy

Microscopy remains the fundamental first-line method for detecting acid-fast bacilli (AFB) in clinical samples. ZN staining and fluorescent microscopy (FM) using auramine or auramine-rhodamine dyes specifically target the mycolic-acid-rich cell wall of mycobacteria. Because this acid-fast property is unique to mycobacteria, AFB microscopy can reliably distinguish NTM and *M. tuberculosis* (MTB) from all other non-acid-fast bacteria, which do not retain these stains. This makes ZN and FM useful in differentiating mycobacteria (AFB-positive) from Gram-negative and Gram-positive organisms, which appear completely negative on the stains. However, since both MTB complex (MTBC) and NTM organisms share the same acid-fast characteristic, microscopy cannot distinguish TB from NTM, even though it readily separates them from non-AFB bacteria.

The performance of these methods has been extensively evaluated in multiple studies. One study demonstrated that both ZN and auramine-based FM are effective for AFB detection and consistently differentiate acid-fast organisms from other bacterial contaminants, confirming the specificity of microscopy for mycobacteria (29). Some studies also identified that both TB and NTM smears appear identical under the microscope, reinforcing that species-level differentiation is not achievable at this stage. Sputum smear microscopy readily detects AFB and therefore identifies mycobacteria vs. other bacterial pathogens, but cannot separate NTM from MTB, which can lead to misdiagnosis if smear microscopy is used alone (30). It has been further emphasized through high specificity of FM in detecting AFB, reinforcing its reliability in ruling out non-mycobacterial organisms in routine screening (31). This has confirmed that both ZN and LED-FM microscopy detect only AFB-positive organisms and no other bacteria, although they lack the ability to discriminate between species of mycobacteria (32). FM has a sensitivity exceeding 70% for TB and over 50% for NTM, again confirming its ability to detect mycobacteria but not distinguish TB from NTM (33). As per laboratory practices, microscopy works well for quick screening, but for final diagnosis and identification of the exact NTM species, advanced lab methods are needed.

### 4.2 Culture

Culture remains the gold standard for the detection and confirmation of NTM because it allows viable organisms to grow, enables species identification, and supports downstream tests such as molecular assays, sequencing, and drug susceptibility testing. Both solid media (Löwenstein-Jensen) and liquid culture systems, particularly the BD BACTEC™ MGIT 960, are widely used in clinical and environmental microbiology. NTM exhibits slow and variable growth rates, frequently requiring 2–8 weeks of incubation depending on the species, and cultures often suffer from high contamination rates, especially when using solid media or liquid media without selective supplements (34). Some studies also demonstrated that traditional MGIT and LJ culture recovered only 32.8% of NTM isolates and showed contamination rates as high as 46%−77%, largely due to the effect of sample flora and the inhibitory effect of NALC-NaOH used during decontamination, which can suppress the growth of fastidious NTM species (34).

Despite the drawbacks, culture remains superior to many molecular approaches for basic detection. Culture outperformed culture-independent sequencing and 16S-based methods in recovering NTM from cystic fibrosis airway samples, emphasizing that viable culture is essential when DNA amplification fails or microbial abundance is low (35). Liquid culture systems such as MGIT960 offer significant strengths, including automated detection, higher sensitivity, and faster time-to-positivity. MGIT can reliably detect *M. chimera* and *M. saskatchewanense* at inocula as low as 4 CFU/ml, showing both high accuracy and reproducibility across repeated runs, which confirms its suitability not only for clinical samples but also environmental surveillance such as water supply testing (17).

In clinical diagnostics, culture is indispensable for national surveillance programs. It has been demonstrated, using over 112,000 AFB culture results from the United States, that culture remains the central tool for monitoring regional variation in NTM prevalence, identifying *M. avium* complex and *M*. *abscessus* as the dominant species, and highlighting the ongoing importance of culture because many molecular probes still do not detect all clinically relevant NTM species (36). Culture additionally forms the basis for advanced identification techniques. Some studies showed that positive MGIT cultures enable rapid and accurate identification, highlighting that culture is a necessary gateway for reliable downstream testing (37). Despite being slow, susceptible to contamination, and reliant on meticulous sample management, culture is unparalleled in its capacity to recover viable NTM, distinguish between colonization and infection, and furnish material for extensive species-level and genomic study.

### 4.3 *hsp*65 gene-based identification

The *hsp65* gene encodes for the 65-kDa heat-shock protein and is found in all mycobacteria. Moreover, unlike highly conserved targets such as 16S rRNA, it shows substantial sequence differences, making it exceptionally useful for distinguishing closely related NTM species. The unique DNA patterns in the *hsp65* gene work as a reliable marker for identifying different species of NTM (38). The most common method is *hsp65*-PRA (polymerase chain reaction restriction analysis), which further amplifies a 441-bp fragment and uses enzyme digestion to create patterns that identify species (39). This technique is fast, cheap, and can be done in regular labs, allowing multiple clinical samples to be processed at the same time. The *hsp65*-PRA method gives accurate species identification in just one test (40). Moreover, it works well with samples from both lung and non-lung sources.

Tools such as Gelcompar software have been used with *hsp65* to improve pattern interpretation and reduce misclassification (41). This sequencing successfully resolved species complexes that are indistinguishable by 16S rRNA sequencing, particularly *M*. abscessus, *M. chelonae, M. simiae*, and *M. gordonae* (42, 43). In addition to its discriminatory power, *hsp65* sequencing is essential in studies requiring phylogenetic analysis, epidemiological tracing, or confirmation of mixed infections, as illustrated by the construction of maximum likelihood phylogenetic trees based on *hsp65* sequencing (43). Despite its strengths, *hsp*65-PRA may face difficulties when species produce fragments of similar sizes, or when small fragments are difficult to differentiate, and mutation-altering restriction sites can occasionally produce ambiguous patterns. It is not always possible to distinguish closely related nontuberculous mycobacterial species using the *hsp*65 gene-based method. Reliance on hsp65 alone can lead to unclear species identification within some phylogenetic complexes, according to several studies. For precise species-level identification, *hsp*65 must frequently be combined with other genetic targets or multigene sequencing techniques (44).

## 5 Advanced diagnostics methods for NTM

Advanced tests such as molecular assays, sequencing, line probe assay, MALDI-TOF, mycobacterial interspersed repetitive units-variable number tandem repeat (MIRU-VNTR) and artificial intelligence (AI) have transformed the same way we detect and identify NTM. Moreover, new testing methods for NTM bacteria have made detection much faster and more accurate than old microscopy and culture techniques. These advanced tools have greatly improved how quickly clinicians can identify these infections and specifically differentiate between NTM and MTBC. These methods also help reveal genes and new mutations that are associated with drug resistance (Figure 2). The following sections covers the important aspects needed for proper patient diagnosis and care.

### 5.1 Sequencing (Sanger, NGS, and WGS)

Sequencing is very important for identifying both the NTM and resistance in this bacteria when other testing methods cannot give clear results. In difficult-to-diagnose cases, this method helps clinicians to know exactly which type of NTM is present. As per various studies, Sanger sequencing of gene targets such as *hsp65, rpoB*, and 16S rRNA gives species-level results and is widely used as a reference method in clinical labs for bacterial identification (42, 45). In particular, *hsp65* and *rpoB* genes can properly distinguish between closely related species such as *M. abscessus, M. chelonae, M*. *simiae*, and *M. gordonae*. Further, new fast DNA reading tools such as next-generation sequencing (NGS) and whole genome sequencing (WGS) are very important for studying genes and tracking antimicrobial resistance outbreaks (43, 44, 46, 47). The study further showed that WGS gives better results for understanding how species are related and allows a detailed study of evolution itself, making it very useful for separating MAC species and fast-growing bacteria (Table 1). WGS and mNGS are the main tools for national surveillance programs, and help to confirm species, detect resistance, and track environmental and clinical NTM spread (36, 48, 49).

**Table 1:** Comparison of advanced diagnostic of NTM.

**Diagnostic method**	**Principle/ target**	**Turnaround time**	**Accuracy**	**Strengths**	**Limitations**	**Clinical utility**	**Key references**
Sanger sequencing	16S, hsp65, rpoB	24–48 h	Moderate–high	High specificity	Cannot detect mixed strains	Species ID	(40)
Targeted NGS	Specific gene panels	24–48 h	High	Detects resistance	Cost	Resistance + ID	(44)
Whole genome sequencing	Whole genome	2–5 days	Highest	Comprehensive	Expensive	Surveillance; resistance	(41)
mNGS	Unbiased sequencing	24–72 h	High	Detects rare species	Contamination risk	Difficult cases	(43)
Line probe assay	PCR + hybridization	6–8 h	High	Fast species ID	Limited probe coverage	Routine labs	(34)
Proteomic MALDI-TOF	Protein spectra	Minutes	Moderate	Cheap, fast	Poor for some NTM	Culture confirmation	(40)
Nucleotide MALDI-TOF	Nucleotide profiles	4–6 h	Sensitivity 77.8%, Specificity 92.5%	Species ID direct from sample	Needs database	Rapid clinical ID	(46)
AI-based imaging	CT pattern recognition	Seconds	High (varies)	Automated screening	Needs training data	TB vs. NTM differentiation	(44)
AI-based genomic interpretation	Pattern detection	Seconds	High	Predicts resistance	Data-dependent	Guide therapy	(44)

Although WGS enables prediction of resistance-associated mutations, it may not fully capture phenotypic or inducible resistance mechanisms, underscoring the continued relevance of conventional susceptibility testing (50, 51). Detection of NTM by sequencing-based approaches does not necessarily indicate active disease and must be interpreted in conjunction with clinical and radiological findings (52). The environmental ubiquity of NTM and the lack of standardized analytical thresholds may result in false-positive detection using metagenomic approaches (50, 53).

### 5.2 Line probe assays

Line probe assays (LPAs) are widely used molecular tools that combine PCR amplification with reverse-hybridization to detect and differentiate between NTM species directly from cultures. LPAs target genes such as *hsp65, rpo*B, and 16S-23S ITS, enabling simultaneous identification of multiple species within a single assay (37). The study also demonstrated that LPAs, such as GenoType^®^ CM/AS/NTM-DR, accurately identified species from positive MGIT cultures in hours, significantly reducing turnaround tine compared with conventional sequencing. LPA provides reliable differentiation among clinically important NTM, including MAC, *M*. *kansasii*, and rapid growers, and are especially useful in laboratories lacking sequencing capabilities (54, 55). LPAs have been shown to detect resistance mutations, particularly in *M. abscessus* and *M. avium complex*, assisting in early treatment decisions. However, LPAs are limited by their predefined probe panels, which may not recognize newly emerging or rare species (34). LPAs alone may miss atypical strains that fall outside the assay's probe coverage, making confirmatory sequencing necessary in complex cases (Table 1). These molecular assays are limited to predefined genetic targets and may not reflect the full phenotypic resistance profile (56).

### 5.3 MALDI-TOF

MALDI-TOF MS has transformed clinical microbiology by enabling rapid, inexpensive, and highly accurate identification of bacteria based on protein spectral fingerprints, and it has become increasingly valuable for NTM species identification. Historically, MALDI-TOF was limited by the tough lipid-rich mycobacterial cell wall; some recent protocols involving extended extraction or bead-beating have improved performance (37, 54). Some studies also suggested that MALDI-TOF MS identifies NTM directly from positive MGIT cultures with up to 95% accuracy, offering a major reduction in time and cost compared with sequencing. Finally, it has been confirmed that MALDI-TOF reliably and reproducibly identifies *M. chimera* and *M. saskatchewanense* from both clinical and environmental samples, underscoring its utility beyond clinical microbiology (17, 57). Selective media and improved extraction protocols significantly enhance MALDI-TOF performance by reducing contamination and ensuring higher-quality spectra from cultured isolates (58). Hence, MALDI-TOF MS is now considered a rapid, reliable, and cost-efficient part of modern NTM workflows, particularly when used in conjunction with MGIT culture.

Despite its rapid turnaround time, the performance of MALDI-TOF MS varies between rapidly growing and slowly growing mycobacteria, with reduced accuracy reported for certain species. MALDI-TOF MS has limited ability to reliably differentiate subspecies within the *M. abscessus* complex and therefore cannot be considered a stand-alone diagnostic tool (59). Complementary molecular methods or WGS are often required for clinically relevant subspecies-level identification (60).

### 5.4 MIRU-VNTR

MIRU-VNTR is a standard PCR-based genotyping procedure that has been modified for NTM to address strain-level diversity and epidemiological trends (24). It generates numeric allelic profiles across multiple VNTR loci, enabling easy inter-laboratory comparison and high epidemiological resolution (61). It has high discriminatory power and reproducibility, ability to produce portable numeric codes for databases, ease of PCR-based processing, and suitability for strain diversity studies (62). Several studies confirm its strong discriminatory power, high reproducibility, ease of use, and cost-effectiveness, making it suitable for routine surveillance. Research from Brazil also demonstrates that MIRU-VNTR identifies clusters of ≥85% similarity and performs well alongside RFLP (54). Similarly, its simplicity and low DNA requirement were highlighted during MAC genotyping in Argentina (63, 64). It may also overestimate or underestimate genetic relationships because of homoplasy and instability of repetitive elements, as shown in MAC studies. Therefore, combining MIRU-VNTR with complementary methods such as spoligotyping or sequencing is recommended for higher accuracy.

### 5.5 Luciferase reporter mycobacteriophage (LRM)–based assays

Luciferase reporter mycobacteriophage (LRM)–based assays represent an advanced phenotypic diagnostic approach that enables rapid detection of viable mycobacteria based on metabolic activity rather than visible growth (65). When these modified phages infect metabolically active mycobacterial cells, they release a luminescent signal and carry luciferase reporter genes, which enables results to be obtained in 24–48 h (66, 67). Rapid drug susceptibility testing, such as the identification of inducible macrolide resistance in the *M. abscessus* complex, has benefited greatly from LRM-based assays (68). These assays greatly shorten diagnostic turnaround times and may enable earlier therapeutic decision-making by avoiding the lengthy incubation times necessary for traditional culture-based techniques. However, their application is currently limited by restricted availability, the requirement for viable organisms, and variable phage infectivity across different NTM species (67). Additionally, the lack of assay standardization and limited clinical validation across diverse laboratory settings remain important challenges. LRM-based assays are best considered complementary tools within reference laboratories rather than stand-alone diagnostic methods at present.

### 5.6 Artificial intelligence

AI is becoming a new tool that helps researchers to find and classify NTM diseases through better imaging and laboratory work. AI-driven systems help clinicians to read medical tests and X-rays automatically, and they can identify different types of diseases. It is the same pattern recognition technology used for all these tasks. AI-assisted microscopy has detected AFB much better than manual checking, giving the same improved results with less human error and faster screening (33). As per recent studies, AI computer programs can tell the difference between NTM lung disease and TB very well using CT scan images (54). This helps clinicians and researchers to find the right disease early and avoid a wrong diagnosis in patients with long-term lung problems (54). In addition, machine learning models integrate MALDI-TOF spectra, resistance predictions and genomic datasets, enabling automated and rapid species identification (36). AI analytics with genetic data are the same core tools used in modern surveillance programs to recognize patterns, group disease cases, and predict regional NTM trends. AI tools need good quality data and proper checking, but they make diagnosis faster and more accurate. Further, these tools will become more common in clinical work as datasets grow larger and the technology itself improves.

## 6 Future prospects of NTM diagnosis

Future developments in NTM research and management will rely heavily on integrating genomics, immunology, and novel therapeutics. Advanced diagnostic strategies, including expanded use of WGS and highly sensitive molecular assays, are expected to improve species differentiation, resistance detection, and outbreak tracing (69). Increasing evidence of person-to-person transmission in specific settings, especially with *M. abscessus*, highlights the need for stronger infection-control policies and genomic surveillance systems (23). Future management will also benefit from host-directed therapies, including strategies that target immune pathways such as IFN-γ, IL-12, and TNF-α, which play critical roles in susceptibility and disease progression (69). Therapeutically, novel antimicrobials, repurposed agents, and bacteriophage therapy—shown effective particularly for rough morphotypes—are anticipated to provide alternatives for multidrug-resistant and refractory disease (69). Improved airway clearance methods, personalized regimens based on strain-level virulence markers, and optimized nutritional and comorbidity management are expected to enhance patient outcomes (69). Global environmental surveillance may clarify rising NTM prevalence and help predict geographic hotspots (23). Future progress will depend on combining molecular precision, host-specific insights, and innovative therapies to achieve earlier diagnosis, individualized treatment, and reduced disease burden.

## 7 Conclusion

The growing global incidence of NTM disease highlights the critical need for rapid, accurate, and standardized diagnostic approaches that can adapt to the expanding diversity of species, complex drug-resistance profiles, and rising clinical impact. While traditional diagnostic methods remain important, they are increasingly inadequate in an era where species-level identification and early detection of resistance are essential for effective treatment. Recent advances in molecular diagnostics, such as *hsp65/rpoB* sequencing, LPAs, MALDI-TOF MS, NGS, and WGS, have revolutionized the field by drastically reducing diagnostic turnaround times and providing exceptional discrimination among closely related species such as *M. abscessus, M. chelonae*, and members of the MAC complex. The integration of AI is further transforming diagnostics, enabling automated smear analysis, imaging-based differentiation of TB and NTM lung disease, and predictive modeling using genomic and proteomic data. Despite these innovations, persistent challenges remain, including inconsistent laboratory practices, limited detection of emerging species, high costs of advanced platforms, and the lack of globally unified diagnostic standards. Overcoming these barriers will require coordinated international action to enhance surveillance, broaden reference databases, validate AI-assisted workflows, and develop species-specific drug susceptibility testing and reporting guidelines. Despite advances in molecular diagnostics, antimicrobial susceptibility assessment for NTM remains complex and cannot rely on genotypic data alone. Integration of laboratory results with clinical judgment remains essential for appropriate therapeutic decision-making. Also, in accordance with the ATS/ERS/ESCMID/IDSA clinical practice guidelines, advanced diagnostic tools for nontuberculous mycobacterial infections should be interpreted within an integrated clinical, radiological, and microbiological framework to avoid over-diagnosis and inappropriate treatment. The future of NTM diagnostics lies in the convergence of genomics, proteomics, advanced culture techniques, and AI-powered analytics, paving the way for precision mycobacteriology, where timely, accurate, and personalized diagnosis becomes the foundation for effective clinical care and global readiness against drug-resistant NTM infections.
